# Geraniin extracted from the rind of *Nephelium lappaceum* binds to dengue virus type-2 envelope protein and inhibits early stage of virus replication

**DOI:** 10.1186/s12985-017-0895-1

**Published:** 2017-11-21

**Authors:** Siti Aisyah Abdul Ahmad, Uma D. Palanisamy, Bimo A. Tejo, Miaw Fang Chew, Hong Wai Tham, Sharifah Syed Hassan

**Affiliations:** 1 Virus-Host Interaction Research Group, Jeffrey Cheah School of Medicine and Health Sciences, Monash University Malaysia, Jalan Lagoon Selatan, 47500 Bandar Sunway, Selangor Malaysia; 2 Department of Chemistry, Universiti Putra Malaysia, 43400 Serdang, Malaysia; 3 Faculty of Applied Sciences, UCSI University, No. 1, Jalan Menara Gading, UCSI Heights, 56000 Kuala Lumpur, Cheras Malaysia; 4 Research Centre for Biomedical Sciences, Sunway University, 47500 Bandar Sunway, Selangor Malaysia; 5 Faculty of Pharmacy, SEGI University, 9 Jalan Teknologi, Taman Sains Selangor, PJU 5, Kota Damansara, 47810 Petaling Jaya, Selangor Malaysia; 6 Infectious Diseases and Health Cluster, Tropical Medicine and Biology Platform, Monash University Malaysia, Bandar Sunway, 47500 Selangor Malaysia

**Keywords:** Antiviral, DENV-2 E protein, Geraniin, *Nephelium lappaceum*

## Abstract

**Background:**

The rapid rise and spread in dengue cases, together with the unavailability of safe vaccines and effective antiviral drugs, warrant the need to discover and develop novel anti-dengue treatments. In this study the antiviral activity of geraniin, extracted from the rind of *Nephelium lappaceum,* against dengue virus type-2 (DENV-2) was investigated.

**Methods:**

Geraniin was prepared from *Nephelium lappaceum* rind by reverse phase C-18 column chromatography. Cytotoxicity of geraniin towards Vero cells was evaluated using MTT assay while IC_50_ value was determined by plaque reduction assay. The mode-of-action of geraniin was characterized using the virucidal, attachment, penetration and the time-of-addition assays’. Docking experiments with geraniin molecule and the DENV envelope (E) protein was also performed. Finally, recombinant E Domain III (rE-DIII) protein was produced to physiologically test the binding of geraniin to DENV-2 E-DIII protein, through ELISA competitive binding assay.

**Results:**

Cytotoxicity assay confirmed that geraniin was not toxic to Vero cells, even at the highest concentration tested. The compound exhibited DENV-2 plaque formation inhibition, with an IC_50_ of 1.75 μM. We further revealed that geraniin reduced viral infectivity and inhibited DENV-2 from attaching to the cells but had little effect on its penetration. Geraniin was observed to be most effective when added at the early stage of DENV-2 infection. Docking experiments showed that geraniin binds to DENV E protein, specifically at the DIII region, while the ELISA competitive binding assay confirmed geraniin’s interaction with rE-DIII with high affinity.

**Conclusions:**

Geraniin from the rind of *Nephelium lappaceum* has antiviral activity against DENV-2. It is postulated that the compound inhibits viral attachment by binding to the E-DIII protein and interferes with the initial cell-virus interaction. Our results demonstrate that geraniin has the potential to be developed into an effective antiviral treatment, particularly for early phase dengue viral infection.

## Background

The fast spread of dengue pandemic especially in tropical countries is worrisome. Every year, 390 million individuals are estimated to be infected with dengue fever, and the occurrence is growing [1]. The countries with the highest disease burden are in the Asia-Pacific region, South and Central America and the Caribbean [2]. The new WHO guideline classifies dengue as dengue with or without warning signs and severe dengue [3]. Dengue is caused by dengue virus (DENV), which belongs to the family *Flaviviridae* and the genus *Flavivirus* [2, 4]. DENV has four antigenically related serotypes named DENV-1, DENV-2, DENV-3, and DENV-4, each causing similar series of illnesses ranging from asymptomatic to a self-limiting febrile illness, to severe and fatal haemorrhagic disease [5]. DENV is mostly spread through its fundamental vector, the *Aedes aegypti*; which is easily bred in tropical urban areas packed with human. DENV is made up of a single-stranded positive-sense RNA genome, sized about 11,000 nucleotides in length. The genome bears an icosahedral nucleocapsid core surrounded by a lipid envelope, wherein the envelope (E) protein and membrane (M) protein are embedded [2, 6, 7]. The major E protein, which forms the glycoprotein shell of the virus, consists of three β-barrel domains namely; Domain I, Domain II, and Domain III [8]. Domain I contains the N-terminus, Domain II mediates dimerization of E, while Domain III is predicted to be involved in receptor binding and antibody neutralization.

Despite the fast spread of dengue pandemic, there is currently no vaccine or antiviral drugs that can effectively prevent or treat dengue infections [9, 10]. However, extensive research is being conducted to validate the ability of certain natural compounds to inhibit DENV [5, 11, 12]. As in other viruses, DENV has several stages in their replication cycle that can be the target for antiviral drugs design. Natural products are promising sources of novel antivirals as they have a variety of chemical constituents that can inhibit the replication cycle of various types of DNA or RNA viruses [13]. *Nephelium lappaceum* is an evergreen tree which is abundant in tropical countries such as Malaysia, Indonesia, Philippines, Thailand and several other regions of the Southeast Asia. *Nephelium lappaceum* belongs to the family *Sapindaceae* [14] and its fruits are normally eaten raw while the seed and rind are discarded. Geraniin was identified as the major compound found in the extract of *Nephelium lappaceum* rind [15]. Geraniin is an ellagitannin, which belongs to the hydrolysable tannin group and occurs naturally in foods such as raspberries, strawberries, blackberries, pomegranate, almonds, and walnuts [16]. Ellagitannins are compounds with a complex chemical structure and have diverse physical properties that lead to various health benefits [17].

Geraniin is present in different parts of various types of plants and has been shown to exhibit antiviral properties against several types of viruses. The compound isolated from *Phyllanthus urinaria* Linnea was shown to actively suppress HSV-1 and HSV-2 infection at different magnitudes of activity [18]. Notka et al. [19] had shown that geraniin extracted from *Phyllanthus amarus* plant had the ability to effectively inhibit HIV-1 replication. Geraniin was identified as one of the active ingredients from the ethanol extract of *Geranium carolinianum* L., a domestic plant grown in China, to exhibit anti-HBV activity [20]. It was also shown that geraniin could effectively inhibit the human enterovirus 71 replications in RD cells and increased the survival rate of infected mice [21]. To date, there are no reports on the specific use of geraniin to inhibit DENV. However, in a study conducted by Lee et al. [11], it was reported that a cocktail of extracts from four species of *Phyllanthus* showed a strong inhibitory activity against DENV-2 and geraniin was found to be the largest component in the extract.

Dengue has become one of the most important arthropod-borne viral diseases and a major public health concern due to the speed of its spread, together with the escalating seriousness of its complications [22]. Unfortunately, despite the advancement of today’s research and development, not even one anti-dengue drug has been approved. Hurdles such as incomplete understanding of dengue pathogenesis and poor funding in the development of anti-dengue drugs were thought to contribute to its absence [23]. Since dengue is marked as a disease of poverty [21], the cost of a drug is also going to be a main issue. Hence, it is important that the source of antiviral molecules be cost-effective, which can be achieved by selecting easy to synthesize chemical scaffolds, or by unearthing the active natural compounds and choosing those that represent major chemical constituents of suitably available plants [24]. As geraniin used in this study was extracted from the rind of *Nephelium lappaceum*, which is considered a waste product, its usage as a source for anti-dengue drug satisfies the cost-effectiveness requirement of an antiviral drug. Previous reports on geraniin’s antiviral activity against several prominent viruses provides evidence that this compound can be effectively used in antiviral therapeutics. Given the need to find an antiviral against DENV, and geraniin’s potential to be developed as a compound of interest, our study aims to investigate the in vitro inhibition of DENV-2 by geraniin and elucidate its mechanistic behavior.

## Methods

### Preparation of geraniin from *Nephelium lappaceum* L. rind by reverse phase C-18 column chromatography

*Nephelium lappaceum* L. was obtained from Kuala Lumpur, Peninsular Malaysia and plants were authenticated by the Herbarium of the Forest Research Institute of Malaysia (FRIM). Crude ethanolic extract of *Nephelium lappaceum* L. rind was prepared as described by Palanisamy and co-workers. Geraniin was purified from the crude extract by means of reverse-phase chromatography [14]. Crude extract (20 g) was dissolved in a minimum amount of water (40 mL) and loaded onto glass column (250 mm × 50 mm i.d.) packed with 200 g C18 silica (particle size 50 μm, pore size 60 Å). The column was open-tubular and solvent flow rate was maintained by means of vacuum pump attached to vacuum inlet in the column. The column was first eluted with water (300 mL) and then fractions were collected using a step gradient of water and acetonitrile. Solvent system was as follows; water (100%, 400 mL), acetonitrile: water (5:95, 350 mL), acetonitrile: water (10:90, 1000 mL). Finally, the column was eluted with methanol (100%, 500 mL). The silica was cleaned by flushing the column sequentially with dichloromethane (100%, 300 mL), methanol containing a few drops of TFA (100%, 300 mL) and absolute methanol (100%, 300 mL), allowing to dry completely. This enables the column to be reused with fresh crude extract. Purity of geraniin (> 95%) obtained was confirmed using HPLC [25]. For use in cell culture, geraniin was initially dissolved in 0.2% dimethyl sulfoxide (DMSO; Sigma-Aldrich; St. Louis, MO, USA) and diluted in minimum essential medium (MEM) containing 2% fetal bovine serum (FBS) (Gibco®; Life Technologies, Carlsbad, CA, USA) to the required concentration, immediately prior to the experiments.

### Cell culture and virus

Propagation of DENV-2 was conducted in African Green Monkey kidney cells (Vero) (ATCC CCL-81). The cells were maintained at 37 °C with 5% CO_2_ and propagated in MEM supplemented with 10% FBS, HEPES buffer and penicillin/streptomycin (Gibco®). DENV-2 (accession no: AJ556810.1) was kindly provided by Prof. Sazaly AB (University of Malaya, Malaysia). Its titer was expressed as plaque forming unit (PFU) per ml. Virus stocks were stored at -80 °C until use.

### Cytotoxicity assay

Vero cells (5 × 10^4^ cells/ml in 96-well-plates) were treated with various concentrations of geraniin for 24 h at 37 °C in 5% CO_2_. Cell viability (%) was then assessed using MTT (3-(4,5-dimethylthiazol-2-yl)-2,5-diphenyl tetrazolium bromide, Sigma-Aldrich; St. Louis, Missouri, USA) assay as described by Javed et al. [26]. Absorbance was measured at 560 nm (OD_560_) using a microplate reader (Bio-Rad; Hercules, CA, USA). All experiments were performed in triplicates and repeated three times.

### Plaque reduction assay

Plaque reduction assay was carried out under two different conditions according to Meneses et al. [27], with minor modifications.

1. Incubation of geraniin with DENV-2 before adsorption: Vero cells (2 × 10^5^ cells/ml in 24-well-plate) were incubated to at least 95% confluency. DENV-2 suspension, with approximately 90–100 plaques per well, was mixed with or without varying concentrations of geraniin. This suspension was incubated at 37 °C for 1 h in MEM containing 2% FBS, before being adsorbed onto cells at 37 °C for 1 h. The mixture was removed and replaced with overlay medium containing MEM with 1% FBS, HEPES, antibiotics and 1% carboxymethyl-cellulose (CMC; Sigma-Aldrich). After a 5-day incubation, the cells were fixed with 10% formalin, followed by staining with 1% crystal violet and plaques counted. The percentage of plaque inhibition compared with controls without geraniin, was calculated according to Cheng et al. [28]. The minimal concentration of geraniin required to reduce 50% plaque number (IC_50_) was deduced from the dose-response curve using GraphPad Prism 5 software (GraphPad Software, Inc.; La Jolla, CA 92037, USA).
2. Incubation of geraniin with cells before adsorption: Vero cells (2 × 10^5^ cells/ml in 24-well-plate) were incubated to at least 95% confluency. The cells were incubated with or without varying concentrations of geraniin at 37 °C for 1 h. Geraniin was then washed off from the plate using PBS and cells were infected with DENV-2 suspension, with approximately 90–100 plaques per well, by adsorption at 37 °C for 1 h. The plate was washed and medium replaced with overlay medium. After 5 days, the cells were fixed and stained using the same procedure described above and plaque number counted.

### Virucidal assay

This assay was conducted to investigate the virucidal effect of geraniin on DENV-2, according to Cheng et al. [27], with minor modifications. Briefly, a virus suspension containing 6.74 × 10^4^ PFU/ml DENV-2 was mixed with or without various concentrations of geraniin for 6 h at 26 °C (room temperature). The samples were subjected to a ten-fold serial dilution, titrated onto confluent monolayer Vero cells (in 24-well plate) and adsorbed at 37 °C for 1 h. The mixture was removed and replaced with overlay medium. After a 5 day incubation, the cells were fixed with 10% formalin followed by staining with 1% crystal violet, and plaques formed were counted. The residual infectivity of each sample was expressed as PFU/ml.

### Attachment assay

This assay was conducted according to a published procedure by Cheng et al. [27], with minor modifications. Briefly, Vero cells were grown in 24-well-plate and pre-chilled at 4 °C for 1 h. Cells were infected with DENV-2 exhibiting 90–100 plaques per well in the absence or presence of various concentrations of geraniin at 4 °C for 3 h. After washing with PBS for three times, the cells were overlaid with overlay medium and incubated at 37 °C for 5 days before being fixed and stained. The percentage of plaque inhibition was estimated.

### Penetration assay

This assay was conducted according to a published procedure by Cheng et al. [27], with minor modifications. Briefly, Vero cells were grown in a 24-well-plate and pre-chilled at 4 °C for 1 h. Cells were infected with DENV-2 exhibiting 90–100 plaques per well and incubated at 4 °C for another 3 h, to allow the attachment of DENV-2 toward cell monolayer. After 3 h, geraniin was added to the wells, with the exception of control, and infected cells were incubated at 37 °C for maximal viral penetration. At every 10 min interval, infected cells were treated with PBS, pH 3 for 1 min to inactivate un-penetrated virus. The acidic media was neutralized using PBS, pH 11, which was then removed and replaced with overlay medium. After further incubation at 37 °C for 5 days, the cells were fixed and stained and its plaque inhibition estimated.

### Kinetics of DENV-2 RNA synthesis by quantitative RT-PCR

Vero cells in 24-well-plate were infected with 6.74 × 10^4^ PFU/ml DENV-2. After a 1 h adsorption at 37 °C, the viral inoculum was removed and MEM supplemented with 2% FBS was added and further incubated at 37 °C. At selected intervals of post-infection, total intracellular viral RNA was extracted using TRI Reagent® Solution (Molecular Research Center, Inc.; Cincinnati, OH, USA) as instructed by the manufacturer. For quantitative RT-PCR (qRT-PCR), a two-step protocol was employed. First, cDNA was synthesized using Maxima First Strand cDNA Synthesis Kit (Fermentas, Thermo Fisher Scientific Inc.; Waltham, MA, USA); second, amplifications were carried out using KAPA SYBR® FAST qPCR Master Mix (KapaBiosystems; Woburn, MA, USA) with the primer pairs designed from conserved region of DENV-2 E gene: forward 5′-GGCCTCGACTTCAATGAGATGG-3′ (1482–1503), reverse 5′-CCTGTTTCTTCGCATGGGGAT-3′ (1639–1659 complement) using ABI Step One and Step One Plus Real Time PCR Systems (Applied Biosystems; Foster City, CA, USA). Cycles consisted of an initial incubation step at 94 °C for 2 min, 40 cycles at 94 °C for 30 s, 60 °C for 30 s, and 72 °C for 30 s, and a melting curve analysis cycle. Absolute quantification of DENV-2 viral load was determined using the standard curve, generated from 10-fold serial dilutions of a plasmid containing DENV-2 gene. All determinations were performed in technical triplicates.

### Time-of-addition assay

This assay was modified from the procedure published by Daelemans et al. [29]. Vero cells in 24-well-plate were infected with 6.74 × 10^4^ PFU/ml DENV-2. After a 1 h adsorption at 37 °C, the excess unbound virus was removed and replaced with MEM supplemented with 2% FBS. The compound, 7-deaza-2′-C-methyladenosine (Santa Cruz Biotechnology, Inc.; Dallas, Texas, USA), which inhibits DENV NS5 polymerase at a later stage of the virus life cycle [30], was used as a positive control in this study. Geraniin (210 μM) or 7-deaza-2′-C-methyladenosine (100 μM) were added at different time intervals (0, 2, 4, 8, 12, 14, 16, 24 h) after infection. At 48 h post-infection (h p.i.), the cells were collected and intracellular viral RNA extracted for viral load quantification using the two-step qRT-PCR described earlier. The percentage of viral RNA inhibition in treated samples compared with untreated controls were calculated.

### Molecular docking

Docking of geraniin to DENV-2 E protein was conducted using the program AutoDock Vina [31]. The three-dimensional structure of DENV-2 E glycoprotein structure was retrieved from the Protein Data Bank (PDB entry: 1OKE) while the structure of geraniin was retrieved from ChemSpider database (http://www.chemspider.com).

### Production of recombinant E-DIII protein

The DENV-2 E gene was amplified using the DENV-2 as template and cloned in-frame and sequenced for its integrity in pCR® 2.1-TOPO TA vector (Invitrogen™, Life Technologies; Carlsbad, CA, USA). The E-DIII was amplified (forward primer: CTGGTACCCTCAAAGGAATGTCATAC, reverse primer: TCGAATTCTTAACTTCCTTTCTTAAACCA, restriction digestion sites underlined), and cloned in-frame into a pRSET-B protein expression plasmid (Invitrogen™, Life Technologies). This clone was identified as pRSETB-EIII. The DNA of pRSETB-EIII was sequenced for the detection of missense and nonsense mutation. For E-DIII expression, pRSETB-EIII was extracted and transformed into *E. coli* BL21 (DE3) strain. Single survived colony was sub-cultured into 500 mL of Luria-Bertani (LB) broth supplemented with ampicillin (100 μg/mL). The culture was maintained in a shaking incubator (37 °C, 150 rpm) until an OD_600_ of 0.5, this was followed by the addition of IPTG (working concentration of 0.5 mM) to induce E-DIII protein expression and the culture was further incubated for 3 h (30 °C, 150 rpm). The bacterial cells were pelleted, and lysed in HEPES buffer (25 mM) using a cell sonicator probe (180 pulse, 3 min). The inclusion bodies were solubilized in urea (8 M) and purified with HisPur Ni-NTA Resin Kit (Thermo Fisher Scientific, Life Technologies) according to the manufacturer’s instructions.

### ELISA competitive binding assay

Saturation binding assay: An ELISA plate was coated with rE-DIII protein dissolved in 0.1 M carbonate-bicarbonate buffer (pH 9.5), and incubated overnight at 4 °C. The plate was washed with TBS containing 0.05% Tween 20 (TBST) and blocked with 1% BSA in TBS (BTBS) at room temperature for 3 h. After washing, the plate was incubated at room temperature for 2 h with serial dilutions of the DENV E-D3 monoclonal antibody, clone 5j122 (MAB8901, Abnova; Taipei, Taiwan) in TBS containing 1% BSA and 0.05% Tween 20 (BTBST). The plate was then washed with TBST and treated with Anti-Mouse IgG (Whole Molecule) Alkaline Phosphatase Conjugate (A5153, Sigma-Aldrich) in BTBST. After a 1 h incubation at room temperature, the plate was washed with TBST and the binding of monoclonal antibody (mAb) to rE-DIII protein was monitored by calorimetric assay using p-Nitrophenyl phosphate (pNPP) as substrate (Sigma-Aldrich). After a 30 min incubation, the absorbance was measured at 405 nm using a plate reader (Bio-Rad). Binding curve and K_d_ (equilibrium dissociation constant) value for the mAb was determined following nonlinear regression analysis on GraphPad Prism 5 software.

Competitive binding assay: An ELISA plate was coated with rE-DIII protein (6.3 μg/ml) dissolved in 0.1 M carbonate-bicarbonate buffer, and incubated overnight at 4 °C. The plate was washed with TBST and blocked with BTBS at room temperature for 3 h. After washing, the plate was incubated for 2 h at 37 °C with various concentrations of geraniin diluted with BTBST. Geraniin was discarded and the plate was washed with TBST. The plate was then incubated at room temperature for 2 h with a single concentration of DENV E-D3 mAb, clone 5j122 in BTBST. The plate was then washed with TBST and treated with Anti-Mouse IgG Alkaline Phosphatase Conjugate in BTBST. After a 1 h incubation at room temperature, the plate was washed with TBST and the binding of mAb to rE-DIII protein was monitored using pNPP as substrate. After a 30 min incubation, absorbance was measured at 405 nm. The competitive binding curve and K_i_ (inhibitory constant: the concentration of a ligand that will bind to half the binding sites at equilibrium in the absence of a competitor ligand) value of geraniin was determined following nonlinear regression analysis on GraphPad Prism 5 software.

### Statistical analysis

The software GraphPad Prism (Version 5.0) was used for all statistical analysis and graphical illustrations. The statistical significance of the difference between mean values was determined by Student’s t-test. The significant difference was considered as *P* < 0.05.

## Results

### Effect of geraniin on DENV-2 plaque formation

**Fig. 1:**
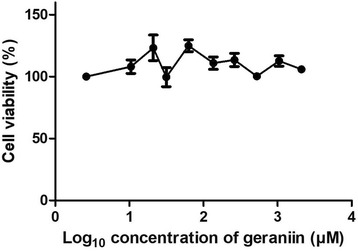
Cytotoxicity assay of geraniin. Geraniin was not toxic to Vero cells even at the highest concentration tested; hence validate its use in the antiviral assays. Data are shown as the mean ± SEM of triplicate from one independent experiment

**Fig. 2:**
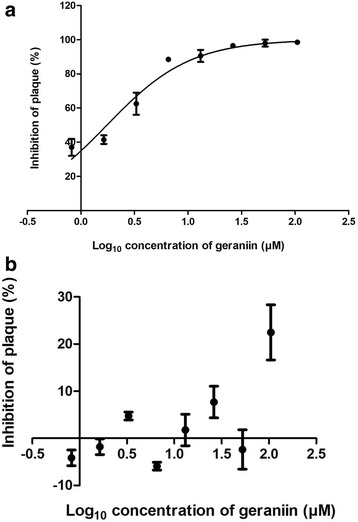
Inhibitory effect of geraniin against DENV-2. **a** A dose response curve showing concentration-dependent inhibition of DENV-2 plaque formation when geraniin was incubated with DENV-2 before adsorption. **b** A dose-response curve indicating that geraniin caused no inhibition towards DENV-2 plaque formation when it was incubated with cells before adsorption. Percentage of plaque inhibition was calculated, and the data was incorporated into the GraphPad Prism 5 software to obtain the IC_50_ values. Data represent mean values ±SEM

MTT assay was conducted to determine the cytotoxicity of geraniin towards Vero cells. Figure 1 shows that geraniin was not toxic to Vero cells even at the highest tested concentration of 2 mM. To determine the antiviral activity of geraniin, plaque reduction assay was carried out under two different conditions; incubation of geraniin with DENV-2 before adsorption and incubation of geraniin with cells before adsorption. Compared with control (without geraniin), plaque formation was significantly reduced when geraniin was incubated with DENV-2 before adsorption. The percentage of plaque inhibition was calculated and dose-response curve generated from the data obtained. Figure 2a shows that when geraniin was incubated with DENV-2 before adsorption, DENV-2 plaque formation was inhibited in a dose-dependent manner, with an IC_50_ of 1.75 μM. However, no inhibition in plaque formation was observed when geraniin was incubated with cells before adsorption (Fig. 2b).

### Mode-of-action of geraniin

**Fig. 3:**
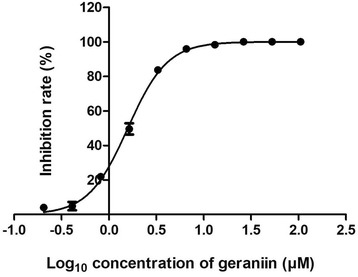
Effect of geraniin on the attachment of DENV-2 to Vero cells. Vero monolayer was pre-chilled at 4 °C for 1 h. DENV-2 was inoculated to Vero monolayer in the absence (virus control) or presence of geraniin and incubated at 4 °C for another 3 h. The dose-response curve indicates the inhibition of DENV-2 attachment to Vero cells by geraniin. Data represent mean values ±SEM

**Fig. 4:**
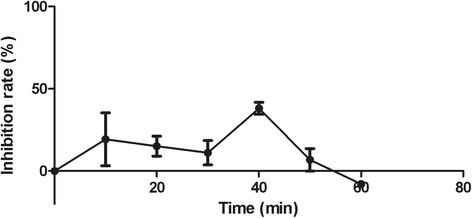
Effect of geraniin on the penetration of DENV-2 into Vero cells. Vero monolayer was pre-chilled at 4 °C for 1 h and then infected with DENV-2 at 4 °C for another 3 h. After 3 h incubation, geraniin was added. At 10 min interval, extracellular virus was killed by PBS at pH 3. PBS at pH 11 was then added to neutralize acidic PBS. The graph of percentage of DENV-2 plaque inhibition was plotted against time taken for penetration. Data represent mean values ±SEM

Although the plaque reduction assay indicated that geraniin was a DENV-2 inhibitor, it was unable to provide information on the mode-of-action of this compound. Therefore, further studies were conducted to determine at which stage geraniin affected the viral life cycle. Virucidal assay was carried out to investigate the effect of varying geraniin concentration on viral residual infectivity. Residual infectivity represents the amount of virus that was not disrupted by geraniin, and hence can still infect cells and replicate. As shown in Table 1, geraniin reduced the infectivity of DENV-2 up to 10^2^ PFU/ml at a concentration of 3.28 μM. Geraniin at concentrations lower than 1.64 μM had no virucidal effect on DENV-2. The virucidal assay confirmed that geraniin reduced the infectivity of DENV-2 in a dose-dependent manner. The attachment assay, on the other hand, was carried out to investigate the effect of geraniin on the attachment of DENV-2 to Vero cells. Figure 3 shows that DENV-2 attachment was significantly inhibited by geraniin in a dose-dependent manner, with a 100% inhibition at the concentration of 26.3 μM. The penetration assay was used to investigate the ability of geraniin to inhibit attached DENV-2 from entering cells. Figure 4 shows that geraniin did not have a significant effect on viruses already attached to cells, and inhibited viruses only in the range of 5–40%.

**Table 1:** Effect of varying geraniin concentrations on DENV-2 residual infectivity

Concentration of Geraniin (μM)	Residual Infectivity (PFU/ml)
0	5.18 × 10^5^
0.41	5.15 × 10^6^
0.82	5.55 × 10^7^
1.64	3.93 × 10^7^
3.28	4.95 × 10^5^
6.57	5.20 × 10^4^
13.13	5.75 × 10^4^
26.26	5.55 × 10^4^
52.52	5.78 × 10^4^
105	3.88 × 10^4^

Virucidal effect of geraniin on DENV-2 was studied by quantifying the infectivity of DENV-2 that remained after it has been exposed to geraniin. DENV-2 with a titer of 6.74 × 10^6^ PFU/ml was used on varying geraniin concentrations for 6 h at 26 °C and the residual infectivity determined

### Geraniin affects early stages of DENV-2 replication

**Fig. 5:**
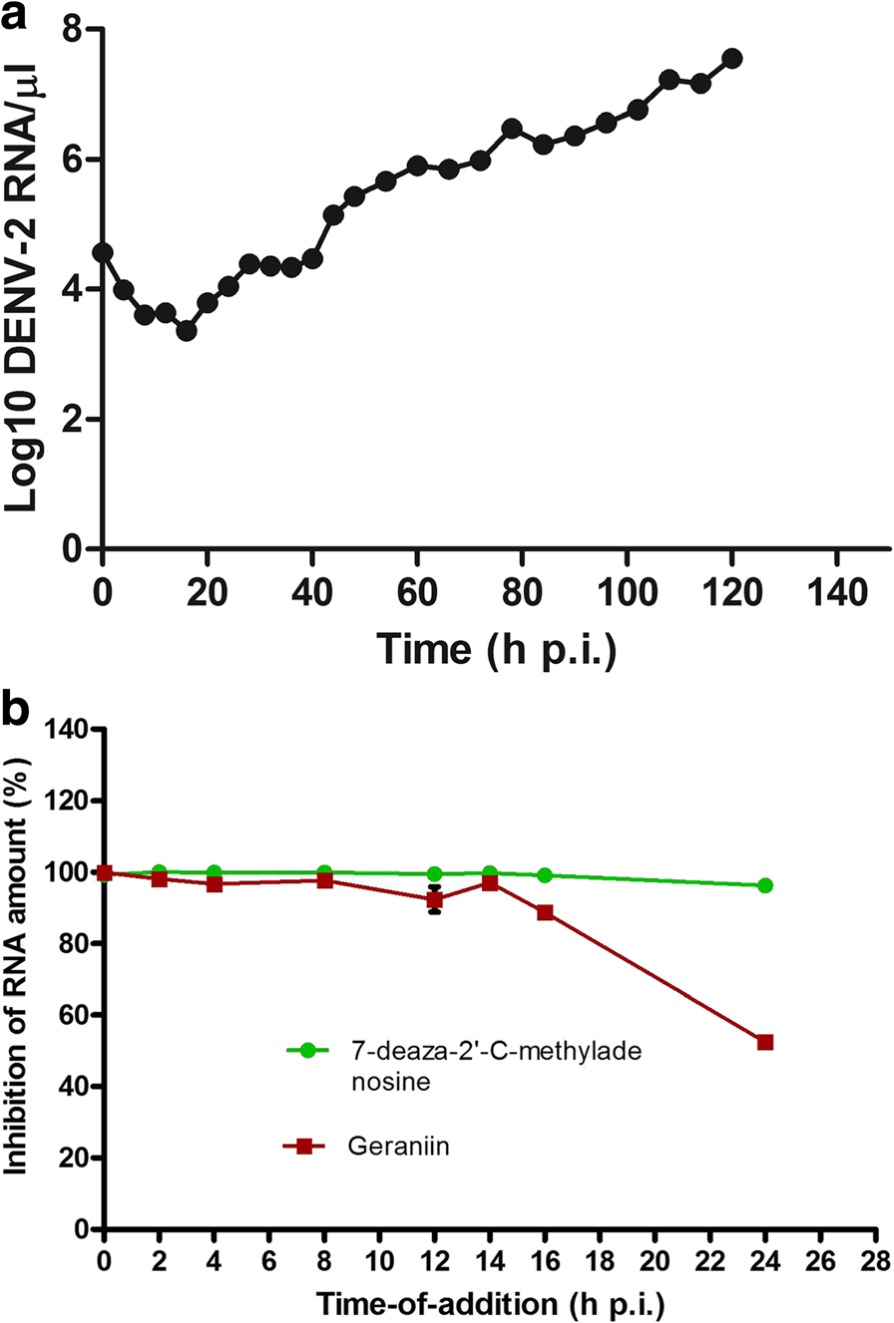
Effect of geraniin on the time of addition. **a** DENV-2 Replication in Vero cells. Vero cells were infected with 6.74 × 10^4^ PFU/ml DENV-2. At selected intervals, intracellular RNA was isolated and DENV-2 RNA levels were quantified using SYBR Green dye-based qRT-PCR assay. Plotted absolute number of viral RNA copy in log scale per μl of RNA used for qRT-PCR. (**b**) Geraniin or the control compound was added to DENV-2 infected Vero cells at different time points after infection. Addition of compounds was conducted up to 24 h before the cells were harvested at 48 h p.i. Samples were subjected to RNA extraction and later to two-step qRT-PCR. Absolute quantification of viral load in treated samples was conducted using the standard curve method. Percentage of inhibition was calculated based on the amount of viral RNA in treated samples compared with untreated controls. Geraniin was effective in inhibiting DENV-2 RNA synthesis when it was added at earlier time-points post-infection. Data represent mean values ±SEM

Time-of-addition assay was carried out to elucidate the stage at which geraniin affected the viral life cycle. The point where geraniin exerts its inhibitory effect during DENV-2 replication cycle may be explicated by determining the effect of different time-of-addition of geraniin on DENV-2 infected cells. To determine this, the time-course kinetic of DENV-2 replication in Vero cells without geraniin treatment must be known. The kinetic of DENV-2 RNA synthesis in infected Vero cells was examined quantitatively by determining the amount of intracellular DENV-2 RNA using the SYBR Green based qRT-PCR amplification. Our results show that the intracellular DENV-2 RNA can be detected up to 120 h p.i. (Fig. 5a). Within the first 0–16 h p.i., there was no increase in RNA copy number, while a gradual increase was observed from 16 to 40 h p.i., followed by a significant increase at 48 h p.i. The RNA copy for DENV-2 increased from around 10^4^ copies/μl at 40 h to 10^5^ copies/μl of RNA at 48 h, as detected by qRT-PCR. The RNA copy number continued to increase up until the 120th h with a value of about 10^7^ copies/μl of RNA. Henceforth, virus growth and replication during the time-of-addition assay was allowed for 48 h for sufficient virus production. In this assay, geraniin was added to DENV-2 infected Vero cells either during the time of infection or at several time points after infection. From the graph shown in Fig. 5b, a constant inhibition of almost 100% was observed throughout the experiment when control compound (7 deaza-2′-C-methyladenosine) was used; retaining its antiviral activity even when added at 24 h p.i. In contrast, geraniin saw a 100% inhibition when added at 0 h p.i., followed by a significant inhibition of about 90% up until when geraniin was added at 16 h p.i. and dropped to about 50% inhibition when geraniin was added at 24 h p.i. Geraniin treatment therefore markedly reduced DENV-2 replication especially at earlier time points or at early stage of infection.

### Geraniin binds to domain III of E protein

**Fig. 6:**
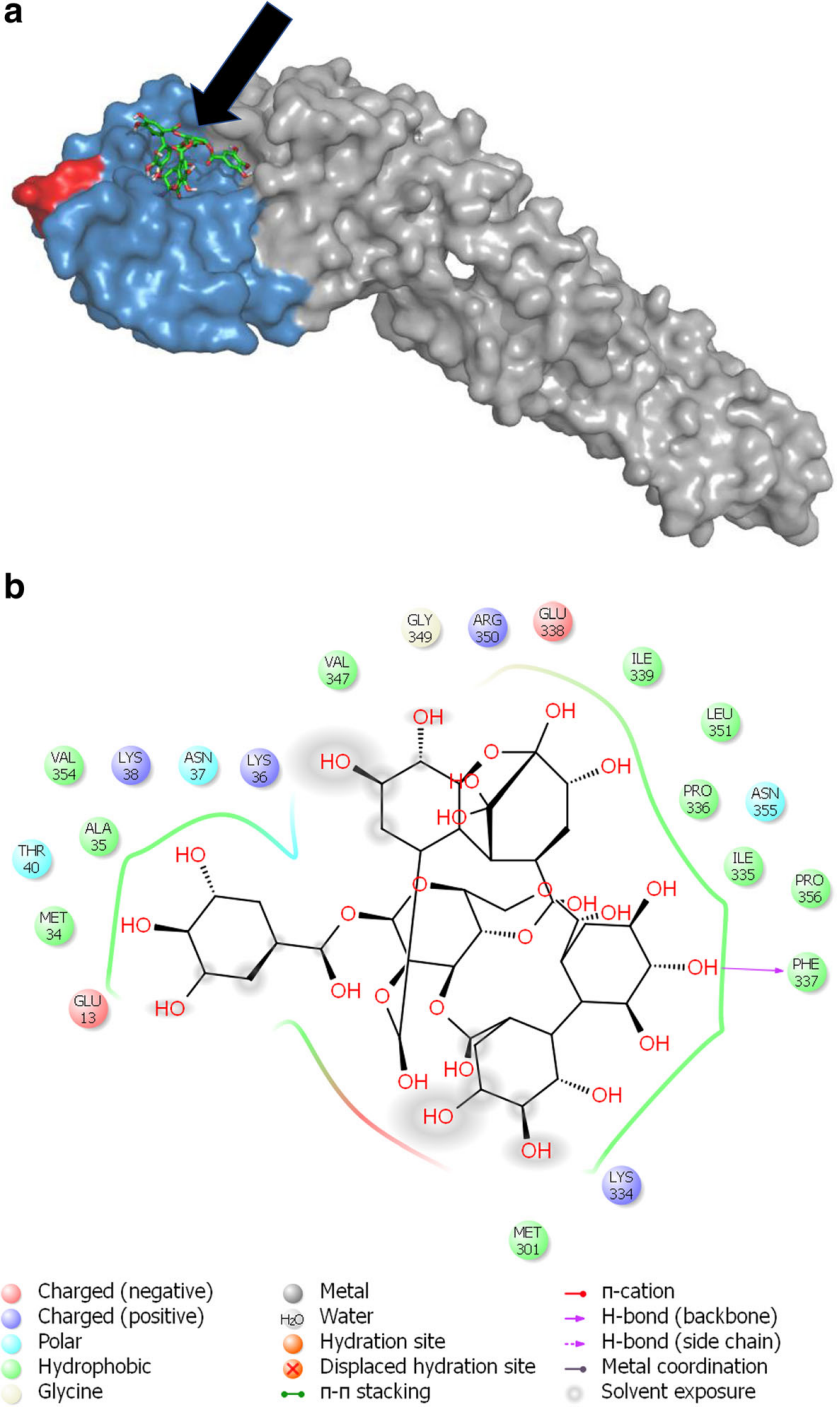
Molecular Docking. **a** Binding pose of geraniin’s molecular structure on DENV-2 E protein (PDB entry: 1OKE). Arrow indicates the molecular structure of geraniin. Domain III is marked blue and a putative receptor-binding loop in domain III is marked red. **b** Interaction of geraniin with the amino acid residues of DENV-2 E protein as shown by docking. Geraniin binds at domain III of E protein which ranges from amino acid residues 296–394, by forming a hydrogen bond with the amino acid residues of the E protein

**Fig. 7:**
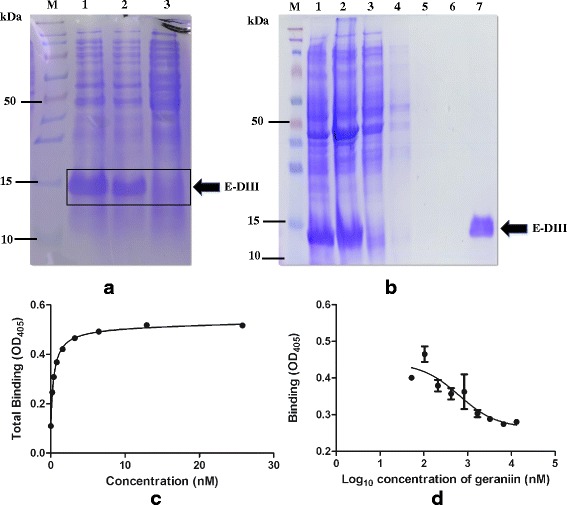
Binding of geraniin to recombinant DENV-2 E Domain III protein. **a** SDS-PAGE gel showing expression of DENV-2 E-DIII protein. Lane M indicates the 10 kDa protein ladder, Lane 1- protein induced with IPTG; Lane 2- protein induced with IPTG; Lane 3- uninduced protein. Size of E-DIII protein is 15 kDa. **b** SDS-PAGE gel showing purification of DENV-2 E-DIII protein. Lane M-10 kDa protein ladder; Lanes; 1-pellet, 2-supernatant, 3-flow-through, 4-wash 1, 5-wash 2, 6-wash 3, 7-eluent. A single band at the expected size indicated that the recombinant protein obtained was pure. **c** The saturation binding curve obtained when recombinant E-DIII protein was coated onto ELISA plate and was allowed to bind to different concentrations of monoclonal antibody, clone 5j122 (MAB8901) before the signal was detected using an AP conjugated secondary antibody. The K_d_ value of the mAb was determined from the curve by nonlinear regression using GraphPad Prism 5 software. **d** The competitive binding curve was obtained through non-linear regression analysis using GraphPad Prism 5 software. The same monoclonal antibody (MAB8901) was used at one constant concentration of 3.33 nM while various concentrations of geraniin was used to test the ability of geraniin to bind to the recombinant E-DIII protein, given that the K_d_ value of the mAb was known. Data represent mean values ±SEM

Docking of geraniin (molecular weight: 952.64 g/mol) was carried out on DENV-2 E protein. Geraniin was shown to bind to domain III of DENV-2 E protein, spanning from amino acid residues 296–394, at a binding affinity of −9.8 kcal/mol. The binding pose of geraniin on DENV-2 E protein is shown in Fig. 6a, while its interaction with the amino acid residues of the protein is shown in Fig. 6b. The binding site of geraniin to E-DIII is between amino acid residues 334–356. To physiologically prove the binding of geraniin to DENV-2 E-DIII protein, a rE-DIII protein was produced. SDS-PAGE confirmed the successfully expression and purification of E-DIII protein (Fig. 7a, b). The single band at the expected size (15 kDa) of the purified E-DIII protein was excised and subjected to in-gel tryptic digestion before it was analyzed on LC-MS/MS to validate its identity and authenticity. Agilent Spectrum Mill search results showed significant hits for DENV-2 polyprotein and the targeted DENV E protein, which confirmed the identity of the purified rE-DIII protein. Binding of geraniin to the rE-DIII protein was then confirmed through a competitive-binding ELISA using a mAb that recognizes recombinant protein corresponding to amino acids 301–400 of DENV E-DIII as the reference analyte. To determine the binding affinity of this mAb for its receptor on our rE-DIII protein, saturation binding assay was conducted. Through this assay, the K_d_ value of the mAb needed to carry out the competitive binding assay, was determined. Figure 7c shows the binding curve of this mAb to the rE-DIII protein. The K_d_ value of the mAb, as determined through nonlinear regression analysis was 0.31 nM. A ligand binding with a K_d_ of 1 nM or less is generally considered to have a high affinity for its receptor, whereas ligands binding with a K_d_ of 1 μM have low affinity [32]. The high affinity of this mAb for the binding site on rE-DIII provides a stronger competition for geraniin as a ligand to the rE-DIII in the competitive binding assay. Having determined the K_d_ value of the reference analyte, the K_i_ value of the competing analyte (geraniin) was then determined through the competitive binding assay. The aim of this competitive assay is to determine the K_i_ or the equilibrium inhibitor constant of geraniin. Geraniin was allowed to bind to the rE-DIII protein coated-plate before incubation with the mAb. It is assumed that geraniin binding to the rE-DIII, will block the mAb from binding to the rE-DIII protein, resulting in a lower ELISA absorbance values. Based on the result in Fig. 7d, it was shown that geraniin does bind to rE-DIII with a K_i_ value of 55.19 nM. As the concentration of geraniin increases, the binding of mAb to the rE-DIII protein decreased. This indicates that geraniin binding to the rE-DIII protein had prevented the mAb from binding to its neutralization site on the rE-DIII protein.

## Discussion

Geraniin was first isolated in 1976 by Okuda et al., from the leaf extracts of *Geranium thunbergii* Sieb. et Zucc.; an official anti-diarrheic and one of the most frequently used folk medicines in Japan [33]. Since then, the occurrence of geraniin has been confirmed in at least 71 different plant species from 26 genera and across 9 families [34]. Here, we assess the effectiveness of geraniin extracted from the rind of *Nephelium lappaceum* against DENV-2. Although DENV has four different serotypes, we chose to work only on DENV-2 since this serotype is more likely to be related to the severe form of dengue. It was observed that patients who are infected with DENV-2 experienced more severe disease and had a higher chance of having dengue haemorrhagic fever (DHF) than those infected with other serotypes [35]. DENV-2 was also responsible for several major dengue outbreaks in Malaysia. DENV-2 emerged as a major serotype in Malaysia in the late 1960s until the early 1970s, and has since persisted in the country, causing major outbreaks that occurred in late 1980s to early 1990s and the subsequent outbreak in late 1990s to early 2000s [36].

The inhibitory potential of geraniin against DENV-2 was first tested using plaque reduction assay, which is considered as the ‘gold standard’ for determining antiviral activity of selected agents against certain viruses [37]. It directly measures the extent to which an antiviral drug inhibits the effect of viral infections in cell culture. Our results from this experimentation implied that geraniin inhibited DENV-2 by affecting either the virus particle itself (virucidal), or by binding to the viral receptors that are involved in the attachment or penetration of virus into cells. However, since no inhibition was observed when geraniin was incubated with the cells before adsorption, it was believed that geraniin’s antiviral activity does not involve the cellular receptors. Adsorption of the virus to cells involves two major steps in the viral life cycle; attachment and penetration. The plaque reduction assay was not able to determine which one of these two steps or both, were involved in the inhibition process. In order to elucidate this, mode-of-action experimentations involving virucidal, attachment, penetration and time-of-addition assays’ were carried out.

Chemical substances are regarded as virucidal agents if they are able to inactivate the extracellular viral particles by damaging the protein coat or penetrating the virion and destroying the viral genome; hence, resulting in decreased infectivity of the virus [38]. The possibility of this occurring was demonstrated using a virucidal assay, where geraniin binding to the virus was shown to have decreased infection and viral replication. Geraniin could either be affecting the structural built-up of the DENV particle, which is made up of C, prM, and E proteins; or it could be affecting the viral genome and destroying the virus from inside out. The questions of whether geraniin exerts its antiviral effect either by entering cells or acting extracellularly remains unanswered [39]. However, the fact that geraniin is a rather large molecule (952.64 g/mol) and being charged, makes it difficult to enter the virion and destroy the genome. It would seem more plausible that geraniin acts extracellularly and at high geraniin concentrations there is an increased probability of its interaction with the virion or its extracellular viral particles. This results in the disruption of infection thus contributing to the decreased infectivity of DENV-2 as seen in our virucidal assay experimentations.

The attachment and penetration assays’ were carried out to study the effect of geraniin on the attachment and penetration of DENV-2 to Vero cells. These assays are dependent on the different temperature requirements of these two initial steps in the viral life cycle. Viral attachment can occur at both 4 °C and 37 °C, while viral penetration can only proceed at 37 °C [3], hence these two steps can be investigated in separate experiments. In the attachment assay, cells were exposed to virus and geraniin at 4 °C, where the virus only attaches to cells, without penetrating them. Plaque formation here was significantly reduced, indicating that geraniin had either prevented DENV-2 attachment to cells or prevented attached DENV-2 from entering cells. The penetration assay confirmed that geraniin did not have a significant effect if present post viral attachment. Our findings propose that geraniin inhibits DENV-2 by disrupting the attachment of the virus with host cellular receptors.

Compounds that can inhibit attachment and prevent entry of pathogenic microbes are appealing because such drugs will not have to gain access to the interior of cells and are therefore expected to display increased bioavailability [40]. In a study carried out by Weaver et al. [41], they proposed the mechanism of action of several tannins against HIV. They showed the tannins ability to significantly block the binding of recombinant HIV coat protein gp120 to its normal cellular receptor CD4. They observed that some of the tannins were required to be present, at the time of infection, in order to exert its potent anti-HIV activity. Similar findings were observed in our study, where a 100% inhibition was observed in the time-of-addition assay upon geraniin’s addition at the time of infection (0 h p.i.). This strengthens our postulation that geraniin exerts its effect by blocking DENV-2 adsorption.

The time-of-addition study suggests the viral life cycle stage that is affected by an inhibitor. In addition, the window of geraniin’s antiviral activity, in relation to the replication cycle of DENV-2, can be defined through this assay [42]. Our findings indicate that geraniin imposed its antiviral effect at the early stage of DENV-2 life cycle, between 0 and 16 h p.i. when viral attachment to its cellular receptors occur prior to its entry into the cytoplasm [43, 44]. This proves that geraniin exerts its antiviral activity towards DENV-2, through the mechanism of inhibiting viral attachment. Since early events in DENV life cycle primarily involves E protein, this protein is suggested the most likely target of inhibition by geraniin. E protein also plays a major role in the control of infection and tropism [43], as well as being involved in several other interactions such as with cellular attachment molecules, which are important for *Flavivirus* entry [7]. These interactions provide promising targets for the design of viral entry inhibitors. Since the attachment of DENV to host cellular receptors is mediated by the E protein [45], we hypothesize that geraniin interferes with the function of E protein during the viral life cycle.

Geraniin is a hydrophobic molecule with a logP value of 3.4. However, when geraniin binds to DENV E protein, the polar and charged interactions overcome the hydrophobic interactions between ligand and protein, due to the presence of multiple hydroxyl groups in the geraniin molecule. Therefore, from a pharmacological point of view, geraniin is a good lead for designing an effective drug in a way that the molecule is sufficiently hydrophobic to cross the cell membrane and in the same time, it has a certain number of polar groups to create significant interactions with DENV E protein. Docking studies were carried out to provide evidence of geraniin’s inhibitory effect on DENV E protein. The host cellular or receptor proteins that bind to the E proteins during attachment have been identified to be within the Domain III region [46]. The docking results showed that the pocket in which geraniin binds to the E-DIII protein is in very close vicinity to the putative receptor-binding loop situated at residue 382–385, which has been associated with the binding process of DENV to receptors [47]. Domain III is an IgC-like module, with ten β-strands. The structure of the four residues of the receptor binding-loop forms a compact solvent-exposed bulge, which is situated between two strands of Domain III. Since geraniin binds near the putative receptor binding loop, there is a possibility that the binding had caused some disturbance on the conformation of the loop and subsequently disrupts the binding of Domain III during DENV infection process.

The docking study serves as an impetus for further investigations into the interaction between geraniin and DENV E protein. To substantiate docking results and provide further evidence of geraniin–DENV-2 E-DIII protein interaction, the rE-DIII protein was produced recombinantly. The addition of geraniin inhibited mAb from binding to the rE-DIII protein site. The findings suggest that either geraniin and the mAb bind to the same site on the rE-DIII protein or that geraniin binds at a different site and changes the conformation of rE-DIII protein hence preventing mAb from binding. Nevertheless, our findings strongly suggest that geraniin binds to DENV-2 E-DIII protein with high affinity with validation from docking studies.

Initial interaction of viruses with target cells is based on specific receptor-ligand interactions between surface structures [48]. For DENVs, the initial binding to target host cells is mediated by binding of the E protein to specific cell surface receptor/s resulting in viral replication and pathogenesis [49]. Molecules that can disrupt virus-host binding and inhibiting the entry of the virus particles into target cells [50] would be considered as very specific inhibitors. The most effective way of preventing viral infection is by blocking the cellular receptors or by using compounds that will bind to the virus receptors’ binding domains, hence preventing the virus from binding to the cellular receptors. The latter approach is preferred since toxicity towards host cells can be avoided.

Overall, our study had proven that geraniin extracted from the rind of *Nephelium lappaceum* has antiviral activity against DENV-2. The mode of its action is either through binding or possibly disruption to the DENV E protein, thus inhibiting viral attachment to cellular receptors during the early stages of the virus life cycle. The binding of geraniin to E-DIII prevents protein-protein interaction between DENV-2 and host cells during infection, thus preventing the attachment and entry process. The exploration of geraniin to be developed into an effective early antiviral treatment for DENV is in line with the current focus on therapeutic strategy for inhibiting enveloped viruses, that is, through the intervention of the viral entry step [29].

## Conclusions

Our study establishes that geraniin has antiviral activity against DENV-2 by inhibiting the mechanism of viral attachment. It most likely shows its effect through the E protein during early stages of the virus life cycle and hence suppresses early viral gene transcription and replication. However, the precise mechanism by which geraniin inhibits DENV-2 gene transcription is still unknown and needs to be further investigated. In vivo experiments to determine the efficacy of geraniin to protect mice from DENV-2 challenge would be beneficial as it can affirm geraniin’s capability to be further developed as an effective anti-dengue agent.

## Abbreviations

ATCC: American Type Culture Collection
CMC: Carboxy-methyl cellulose
CO_2_: Carbon dioxide
DENV: Dengue virus
DENV-1: Dengue virus type-1
DENV-2: Dengue virus type-2
DENV-3: Dengue virus type-3
DENV-4: Dengue virus type-4
DHF: Dengue hemorrhagic fever
E: Envelope
FBS: Fetal bovine serum
h: Hour
HBV: Hepatitis B virus
HEPES: 4-(2-hydroxyethyl)-1-piperazineethanesulfonic acid
HIV: Human immunodeficiency virus
HSV: Herpes simplex virus
IC_50_: Half maximal inhibitory concentration
LCMS/MS: Liquid chromatography tandem mass spectrometry
M: Membrane
M: Molar
mAb: Monoclonal antibody
MEM: Minimum essential medium
MIC: Minimum inhibitory concentration
mRNA: Messenger Ribonucleic acid
MTT: (3-(4,5-Dimethylthiazol-2-yl)-2,5, diphenyltetrazoliumbromide
NaOH: Sodium hydroxide
OD: Optical density
PBS: Phosphate buffered saline
p.i.: Post-infection
PFU: Plaque forming unit
qRT-PCR: Quantitative real time RT-PCR
rE-DIII: Recombinant E domain III protein
RT-PCR: Reverse transcription polymerase chain reaction
WHO: World Health Organization

## References

[1] Bhatt S (2013). The global distribution and burden of dengue. Nature.

[2] Gubler, D.J., Dengue Viruses, in Encyclopedia of Virology (Third Edition), B.W.J. Mahy and M.H.V.V. Regenmortel, Editors. 2008, Academic Press: Oxford. p. 5–14.

[3] World Health Organization. Dengue: guidelines for diagnosis, treatment, prevention and control: New Edition. Geneva; 2009. Available from http://www.who.int/rpc/guidelines/9789241547871/en/. 23762963

[4] Hung S-L (1999). Analysis of the steps involved in dengue virus entry into host cells. Virology.

[5] Whitehorn J, Farrar J (2010). Dengue. Br Med Bull.

[6] Ocazionez RE (2010). Virucidal activity of Colombian Lippia essential oils on dengue virus replication in vitro. Mem Inst Oswaldo Cruz.

[7] Patkar, C.G. and R.J. Kuhn, Development of novel antivirals against Flaviviruses, in New Treatment Strategies for Dengue and Other Flaviviral Diseases. 2008, John Wiley & Sons, Ltd. p. 41–56.

[8] Perera R, Khaliq M, Kuhn RJ (2008). Closing the door on flaviviruses: entry as a target for antiviral drug design. Antivir Res.

[9] Rey FA (2013). Dengue virus: two hosts, two structures. Nature.

[10] Kaptein SJF, Neyts J (2016). Towards antiviral therapies for treating dengue virus infections. Curr Opin Pharmacol.

[11] Parida MM (2002). Inhibitory potential of neem (Azadirachta Indica Juss) leaves on dengue virus type-2 replication. J Ethnopharmacol.

[12] Lee S (2013). Effects of cocktail of four local Malaysian medicinal plants (Phyllanthus spp.) against dengue virus 2. BMC Complement Altern Med.

[13] Jassim SAA, Naji MA (2003). Novel antiviral agents: a medicinal plant perspective. J Appl Microbiol.

[14] Ong PK, Acree TE, Lavin EH (1998). Characterization of volatiles in Rambutan fruit (Nephelium Lappaceum L.). J Agric Food Chem.

[15] Palanisamy UD (2011). Rapid isolation of geraniin from Nephelium Lappaceum rind waste and its anti-hyperglycemic activity. Food Chem.

[16] Ito H (2011). Metabolites of the ellagitannin geraniin and their antioxidant activities. Planta Med.

[17] Lipińska, L., E. Klewicka, and M. Sójka, The structure, occurrence and biological activity of ellagitannins: a general review. Acta Sci. Pol., Technol. Aliment, 2014. 13(3): p. 289–299. 10.17306/j.afs.2014.3.7 24887944

[18] Yang CM (2007). The in vitro activity of geraniin and 1,3,4,6-tetra-O-galloyl-beta-D-glucose isolated from Phyllanthus Urinaria against herpes simplex virus type 1 and type 2 infection. J Ethnopharmacol.

[19] Notka F, Meier GR, Wagner R (2003). Inhibition of wild-type human immunodeficiency virus and reverse transcriptase inhibitor-resistant variants by Phyllanthus Amarus. Antivir Res.

[20] Li J (2008). In vitro and in vivo anti-hepatitis B virus activities of a plant extract from Geranium Carolinianum L. Antivir Res.

[21] Yang Y (2012). Antiviral effect of geraniin on human enterovirus 71 in vitro and in vivo. Bioorg Med Chem Lett.

[22] Phillips ML (2008). Dengue reborn: widespread resurgence of a resilient vector. Environ Health Perspect.

[23] Perry ST, Buck MD, Shresta S (2011). Better late than never: antivirals for dengue. Expert Rev Anti-Infect Ther.

[24] Canard B. Antiviral research and development against dengue virus. 2010 8 August 2012]; Available from: http://www.who.int/tdr/research/ntd/dengue/dengue_full_length_report.pdf.

[25] Perera A (2012). Large scale purification of geraniin from Nephelium Lappaceum rind waste using reverse-phase chromatography. Sep Purif Technol.

[26] Javed T (2011). In-vitro antiviral activity of Solanum Nigrum against hepatitis C virus. Virol J.

[27] Meneses R (2009). Inhibitory effect of essential oils obtained from plants grown in Colombia on yellow fever virus replication in vitro. Ann Clin Microbiol Antimicrob.

[28] Cheng HY, Lin CC, Lin TC (2002). Antiherpes simplex virus type 2 activity of casuarinin from the bark of Terminalia Arjuna Linn. Antivir Res.

[29] Daelemans D (2011). A time-of-drug addition approach to target identification of antiviral compounds. Nat Protoc.

[30] Wang QY (2009). A small-molecule dengue virus entry inhibitor. Antimicrob Agents Chemother.

[31] Trott O, Olson AJ (2010). AutoDock Vina: improving the speed and accuracy of docking with a new scoring function, efficient optimization and multithreading. J Comput Chem.

[32] Davenport A, Russell F, Mather S (1996). Radioligand binding assays: theory and practice. Current Directions in Radiopharmaceutical Research and Development.

[33] Okuda T, Yoshida T, Nayeshiro H (1976). Geraniin, a new ellagitannin from Geranium Thunbergii. Tetrahedron Lett.

[34] Cheng HS, Ton SH, Abdul Kadir K. Ellagitannin geraniin: a review of the natural sources, biosynthesis, pharmacokinetics and biological effects. Phytochem Rev. 2016:1–35.

[35] Vaughn DW (2000). Dengue viremia titer, antibody response pattern, and virus serotype correlate with disease severity. J Infect Dis.

[36] Chee H-Y, AbuBakar S (2003). Phylogenetic investigation of dengue virus type 2 isolated in Malaysia. Dengue Bulletin.

[37] Shors, T., Virus replication cycles, in Understanding viruses (Second edition). 2013, Jones & Bartlett learning: Burlington, MA. p. 46–69.

[38] Galabov, A.S., Virucidal agents in the eve of manorapid synergy*.* GMS Krankenhhyg Interdiszip, 2007. 2(1): p. Doc18. PMC2831485 20200679

[39] Perera A, Ton SH, Palanisamy UD (2015). Perspectives on geraniin, a multifunctional natural bioactive compound. Trends Food Sci Technol.

[40] Seema and S.K. Jain, Molecular mechanism of pathogenesis of dengue virus*:* Entry and fusion with target cell*.* Indian J Clin Biochem, 2005. 20(2): p. 92–103. 10.1007/BF02867407 PMC3453834 23105540

[41] Weaver JL (1992). Prevention of binding of rgp120 by anti-HIV active tannins. Biochem Pharmacol.

[42] Low JS (2011). Narasin, a novel antiviral compound that blocks dengue virus protein expression. Antivir Ther.

[43] van der Schaar HM (2007). Characterization of the early events in dengue virus cell entry by biochemical assays and single-virus tracking. J Virol.

[44] Acosta EG, Talarico LB, Damonte EB (2008). Cell entry of dengue virus. Futur Virol.

[45] Bielefeldt-Ohmann H (2001). Dengue virus binding to human leukocyte cell lines: receptor usage differs between cell types and virus strains. Virus Res.

[46] Hidari KIPJ, Suzuki T (2011). Dengue virus receptor. Tropical Medicine and Health.

[47] Modis Y (2003). A ligand-binding pocket in the dengue virus envelope glycoprotein. Proc Natl Acad Sci U S A.

[48] Chen Y, Maguire T, Marks RM (1996). Demonstration of binding of dengue virus envelope protein to target cells. J Virol.

[49] Mairiang D (2013). Identification of new protein interactions between dengue fever virus and its hosts, human and mosquito. PLoS One.

[50] Chin JF, Chu JJ, Ng ML (2007). The envelope glycoprotein domain III of dengue virus serotypes 1 and 2 inhibit virus entry. Microbes Infect.

